# Dynamic MRI for articulating joint evaluation on 1.5 T and 3.0 T scanners: setup, protocols, and real-time sequences

**DOI:** 10.1186/s13244-020-00868-5

**Published:** 2020-05-19

**Authors:** Marc Garetier, Bhushan Borotikar, Karim Makki, Sylvain Brochard, François Rousseau, Douraïed Ben Salem

**Affiliations:** 1 Department of Radiology, Military Teaching Hospital Clermont-Tonnerre, Rue du colonel Fonferrier, 29240 Brest, Cedex 9 France; 2 Department of Radiology, University Hospital Morvan, Brest, France; 3 Laboratory of Medical Information Processing (LATIM), INSERM-UMR 1101, Brest, France; 4 University of Western Brittany (UBO), Brest, France; 5 University Hospital, Brest, France; 6 IMT Atlantique, UBL, Brest, France; 7 Department of Physical and Medical Rehabilitation, University Hospital Morvan, Brest, France; 8 Department of Paediatric Physical and Medical Rehabilitation, Fondation Ildys, Brest, France; 9 Department of Radiology, University Hospital La Cavale Blanche, Brest, France

**Keywords:** Joints, Motion, Musculoskeletal system, Magnetic resonance imaging

## Abstract

Dynamic magnetic resonance imaging (MRI) is a non-invasive method that can be used to increase the understanding of the pathomechanics of joints. Various types of real-time gradient echo sequences used for dynamic MRI acquisition of joints include balanced steady-state free precession sequence, radiofrequency-spoiled sequence, and ultra-fast gradient echo sequence. Due to their short repetition time and echo time, these sequences provide high temporal resolution, a good signal-to-noise ratio and spatial resolution, and soft tissue contrast. The prerequisites of the evaluation of joints with dynamic MRI include suitable patient installation and optimal positioning of the joint in the coil to allow joint movement, sometimes with dedicated coil support. There are currently few recommendations in the literature regarding appropriate protocol, sequence standardizations, and diagnostic criteria for the use of real-time dynamic MRI to evaluate joints. This article summarizes the technical parameters of these sequences from various manufacturers on 1.5 T and 3.0 T MRI scanners. We have reviewed pertinent details of the patient and coil positioning for dynamic MRI of various joints. The indications and limitations of dynamic MRI of joints are discussed.

## Key points

- A real-time gradient echo sequence depicts functional details of the joint during motion.
- Appropriate and customized patient setup and coil installation inside the MR bore are fundamental for the exploration of joint motion.
- Artifacts due to the inherent joint motion and those related to real-time sequence parameters can be reduced to improve image quality and diagnostic capability.

## Background

Being non-invasive, magnetic resonance imaging (MRI) is widely used in the clinical decision-making process. Static morphological MRI is useful for the diagnosis of musculoskeletal disorders but does not represent the dynamic physiology of joints [1–5]. Dynamic in vivo imaging may provide valuable functional information (qualitative and quantitative) in addition to static imaging; thus, it may help in the selection of an optimal treatment strategy [6]. In vivo imaging of the joint motion can be performed using ultrasonography [7], single or biplanar fluoroscopy [1, 8], computed tomography (CT) [9], and MRI [10]. The role of ultrasonography is limited to the evaluation of the soft tissue around the joint. Fluoroscopy and CT scan modalities are limited to the quantification of bone kinematics and expose the individuals to ionizing radiation. MRI provides anatomical details of bones and soft tissues in static and dynamic acquisitions without exposure to ionizing radiation.

Dynamic MRI sequences were developed as early as 1984 for cardiac imaging [11, 12] and were subsequently applied to the musculoskeletal system to quantify bone motion and joint kinematics [13–15]. Dynamic MRI-based musculoskeletal system evaluations could be significantly different from those performed using static MRI. For example, Muhle et al. showed that dynamic MRI was significantly better than static imaging for the demonstration of patellar tilt angle, particularly at the critical range of patellar instability between 30° and 0° of knee flexion [16]. The existing literature shows no consensus regarding the technical parameters and the use of various sequences for dynamic MRI of joints. This is due to the existence of multiple and custom-built dynamic MRI sequences and the lack of standard/built-in dynamic musculoskeletal MRI sequences from the MRI scanners’ original equipment manufacturers. Dynamic MRI is currently still not used in standard clinical practice for the management of musculoskeletal disorders [17]. Optimal dynamic MRI of the joint requires the following:

1. The adaptation of available dynamic MRI sequences to the shortest acquisition time to enable in vivo imaging of the joint during a single cycle of voluntary motion performed by the patient.
2. The customization of the MRI sequences and the scanning parameters according to the field strength and manufacturer of the MRI scanners.
3. The standardization of the patient setup and radiofrequency (RF) coil positioning for each type of joint and its range of motion (ROM).

As many as eight types of dynamic MRI sequences have been reported in the literature. Of these, real-time sequences provide fast, trigger-free, and multi-slice acquisitions and are most suitable for in vivo joint evaluations in individuals with joint disorders [17]. In the following sections, we focus on real-time sequences and provide relevant details of the following:

1. Real-time sequence parameters used by multiple manufacturers on 1.5 T and 3.0 T MRI scanners.
2. Practical recommendations of patient and coil positioning in the scanner for optimal motion and image acquisition.
3. Perspectives and limitations on the use of dynamic MRI as standard clinical practice.

## Real-time dynamic MRI sequences and parameters

Joint motion imaging in MRI can be acquired by three methods [18]:

- Incremental (quasi-static) acquisition, with a change in the joint position between each acquisition. In this case, it is possible to use the static sequences, with the acquisition time multiplied by the number of sequence repetitions at each position [19–22].
- Motion-triggered acquisition, with the image database reconstructed according to the position of the joint during the acquisition cycle, requiring to repeat the movement several times, including cine-MRI and cine phase-contrast techniques [23, 24].
- Real-time acquisition, allowing acquisition in a few seconds during continuous joint motion, with no repetition required [25–27].

In their systematic review, Borotikar et al. concluded that cine phase-contrast and real-time sequences were the two types of sequences that provided excellent validity and reliability for joint motion evaluation by MRI [17]. Cine phase-contrast sequences provide quantitative data, such as the three-dimensional (3D) pixel velocity in moving structures, coupled with anatomical images. However, image acquisition through these triggered sequences requires the patient to perform repeated joint motions. This leads to an acquisition time of a few minutes, which generates pain and fatigability in patients and in turn introduces averaging error in the acquired images due to the loss of movement reliability [10].

Real-time dynamic MRI, on the other hand, is a fast imaging technique that is mainly based on rapid gradient echo sequences with a flip angle of less than 90° and a significant reduction of the repetition time (TR) lower than the T2 relaxation time. The low TR is responsible for a residual transverse magnetization before the next RF pulse. These sequences are particularly adapted for dynamic joint MRI because of their high temporal resolution, thereby allowing the acquisition of an image in a few hundred milliseconds and the fast repetition of the slices. The entire joint can be covered during a single acquisition, and the relationship between joint structures can be visualized to study normal and pathological joint physiology [26]. Three gradient echo sequences typically available on MRI scanners are suitable for real-time dynamic MRI and have already been used in previous studies to understand in vivo joint biomechanics. These are balanced steady-state free precession (SSFP) sequence [26, 28–33], RF-spoiled sequence [29, 34–36], and ultra-fast gradient echo (UFGE) sequence [5, 37], with different acronyms used by each MRI scanner manufacturer (Table 1).

**Table 1:** Manufacturer acronyms of gradient echo sequences used for real-time joint dynamic MRI

	Balanced SSFP	RF-spoiled	UFGE
GE Healthcare	FIESTA	SPGR	FSPGR
Philips Healthcare	bFFE	T1FFE	TFE
Siemens Healthineers	TrueFISP	FLASH	TurboFLASH

*bFFE* balanced Fast Field Echo, *FLASH* Fast Low-Angle SHot, *FIESTA* Fast Imaging Employing STeady-state Acquisition, *FISP* Fast Imaging with Steady-state Precession, *FSPGR* Fast SPoiled Gradient-Recalled, *TFE* Turbo Fast Echo

**Fig. 1:**
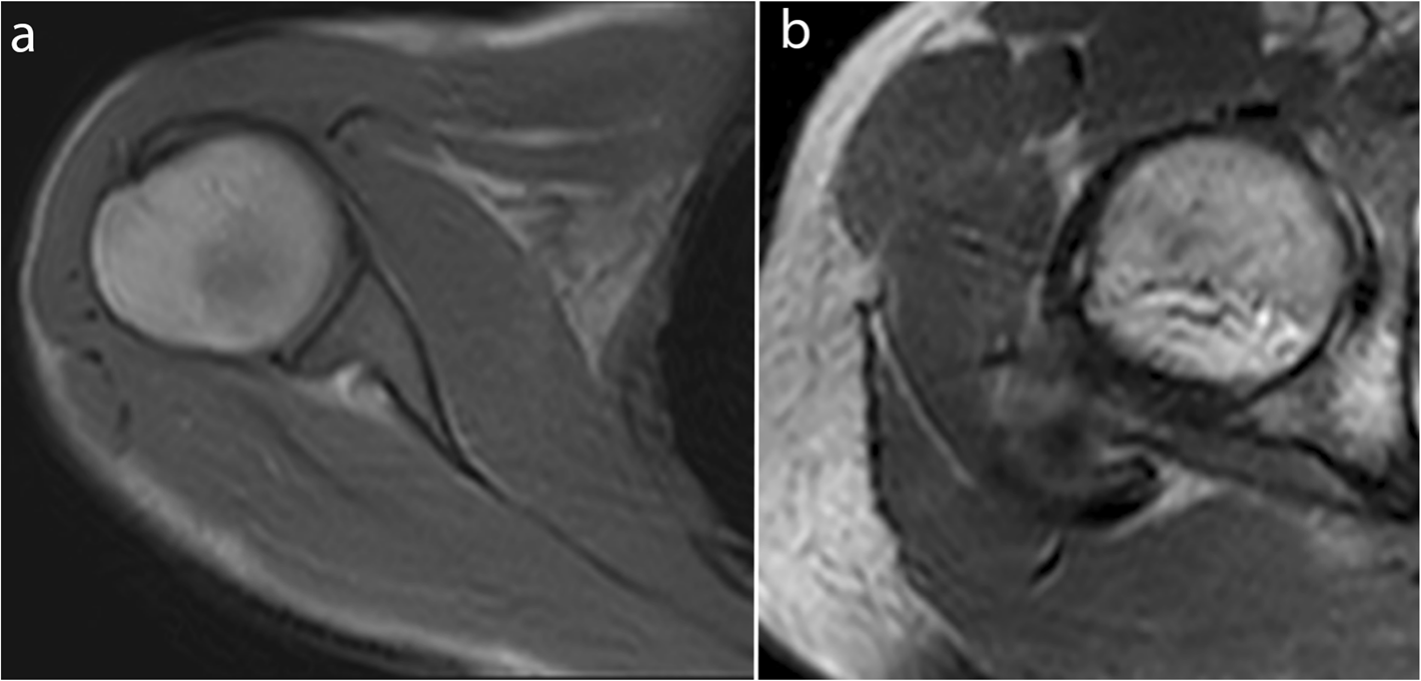
Real-time dynamic MR images obtained with balanced SSFP sequence at 3.0 T. Shoulder in the axial plane in external rotation (**a**) and hip in the axial plane in external rotation (**b**) (TR, 4.2; TE, 2.1; flip angle, 40°; pixel size, 1.37 × 1.83 mm)

**Fig. 2:**
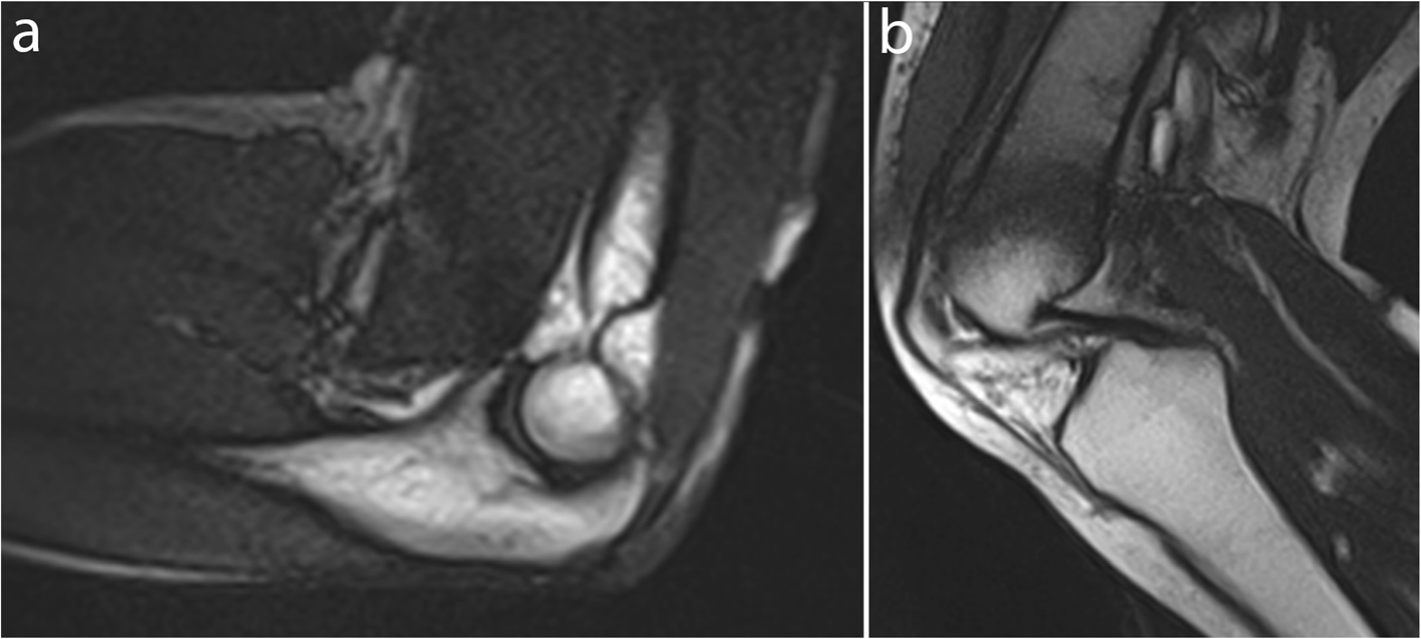
Real-time dynamic MR images obtained with balanced SSFP sequence at 1.5 T. Elbow in the sagittal plane in flexion (**a**) and knee in the sagittal plane in flexion (**b**) (TR, 4.6; TE, 2.3; flip angle, 40°; pixel size, 1.09 × 1.46 mm)

In a balanced SSFP sequence, a steady state between residual transverse and longitudinal magnetization is reached after the administration of a train of RF pulses, with the shortest TR and echo time (TE). All the gradients are fully refocused and balanced symmetrically, thereby allowing an excellent contrast between structures with a high signal-to-noise ratio (SNR), which is independent of TR and less sensitive to motion artifacts [26, 38–40]. This sequence is T2-weighted, with contrast dependent on the T2/T1 ratio of each tissue, which results in a high fat and water signal (Figs. 1 and 2, Movie 1, 2) [41]. In the RF-spoiled sequence, the RF phase modulation at each cycle deletes the residual transverse magnetization for T1 contrast with a TR value usually around 20–30 ms [38, 40]. The UFGE sequence, based on the RF-spoiled gradient echo technique using a small flip angle, allows for the reduction of TR below 10 ms and, thus, reduces acquisition time [40, 42]. A few studies used a post-excitation refocused gradient echo sequence with radial sampling, allowing a T2/T1 contrast and high temporal resolution. However, this sequence is a work-in-progress package only provided by one manufacturer and cannot be proposed in the current practice [43].

Table 2 provides a summary of the sequence parameters reported in the literature since 2010 for real-time dynamic MRI of joints of the limbs with 1.5 T and 3.0 T MRI scanners, based on commercially available balanced SSFP, RF-spoiled, and UFGE sequences, for their implementation in daily practice.

**Table 2:** Sequence parameters reported in the recent literature for real-time joint dynamic MRI sequences at 1.5 T and 3.0 T

	Balanced SSFP		RF-spoiled		UFGE
Field strength	1.5 T [28]	3.0 T [29, 33]	1.5 T [36, 44]	3.0 T [29, 34, 35]	3.0 T [5, 37]
TR (ms)	3.6	3.98–4.71	3.18–7.8	3.13–20.6	2.4–2.7
TE (ms)	1.3	1.75–2.36	1.4–3.3	1.74–3.3	1.2–1.3
Flip angle (degree)	65	46–47	8–20	8–20	10–15
Bandwidth (Hz/pixel)	NR	454–930	200, NR for [44]	990	NR
Pixel size (mm)	1.6	0.94–1.07	1.4–1.56 × 2.88	0.57–1.07	1
Slice thickness (mm)	10	6	10	6–8	4–5
Number of time frames	7	10–60	40, NR for [44]	10–16	30–40
Mean acquisition time/image (ms)	285	475–562	331–500	197–351	31–233

*NR* not reported

**Fig. 3:**
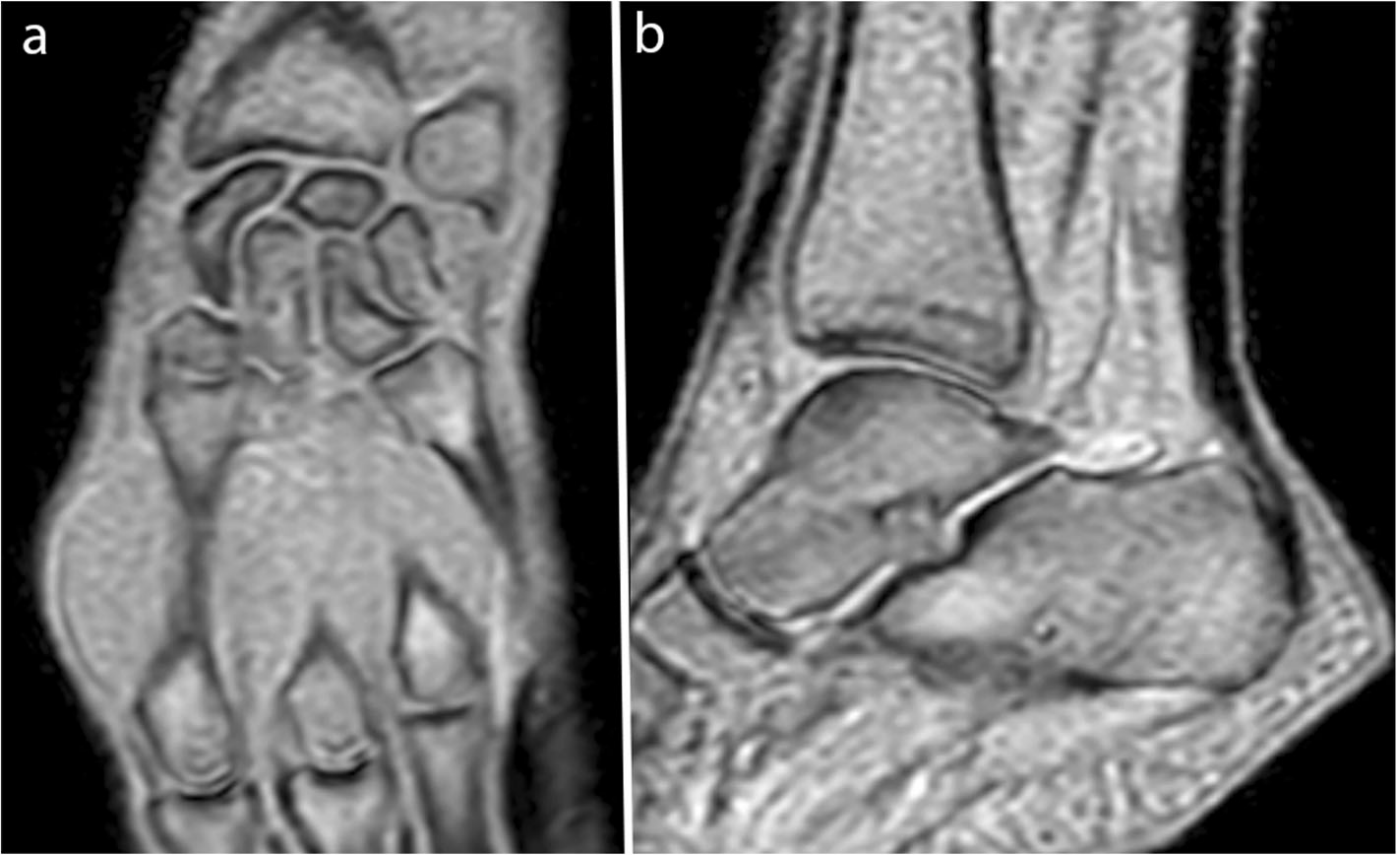
Real-time dynamic MR images obtained with the RF-spoiled sequence at 3.0 T. Wrist in the coronal plane in ulnar deviation (**a**) and ankle in the sagittal plane in plantar flexion (**b**) (TR, 20.6; TE, 1.8; flip angle, 15°; pixel size, 1.3 × 1.67 mm)

**Fig. 4:**
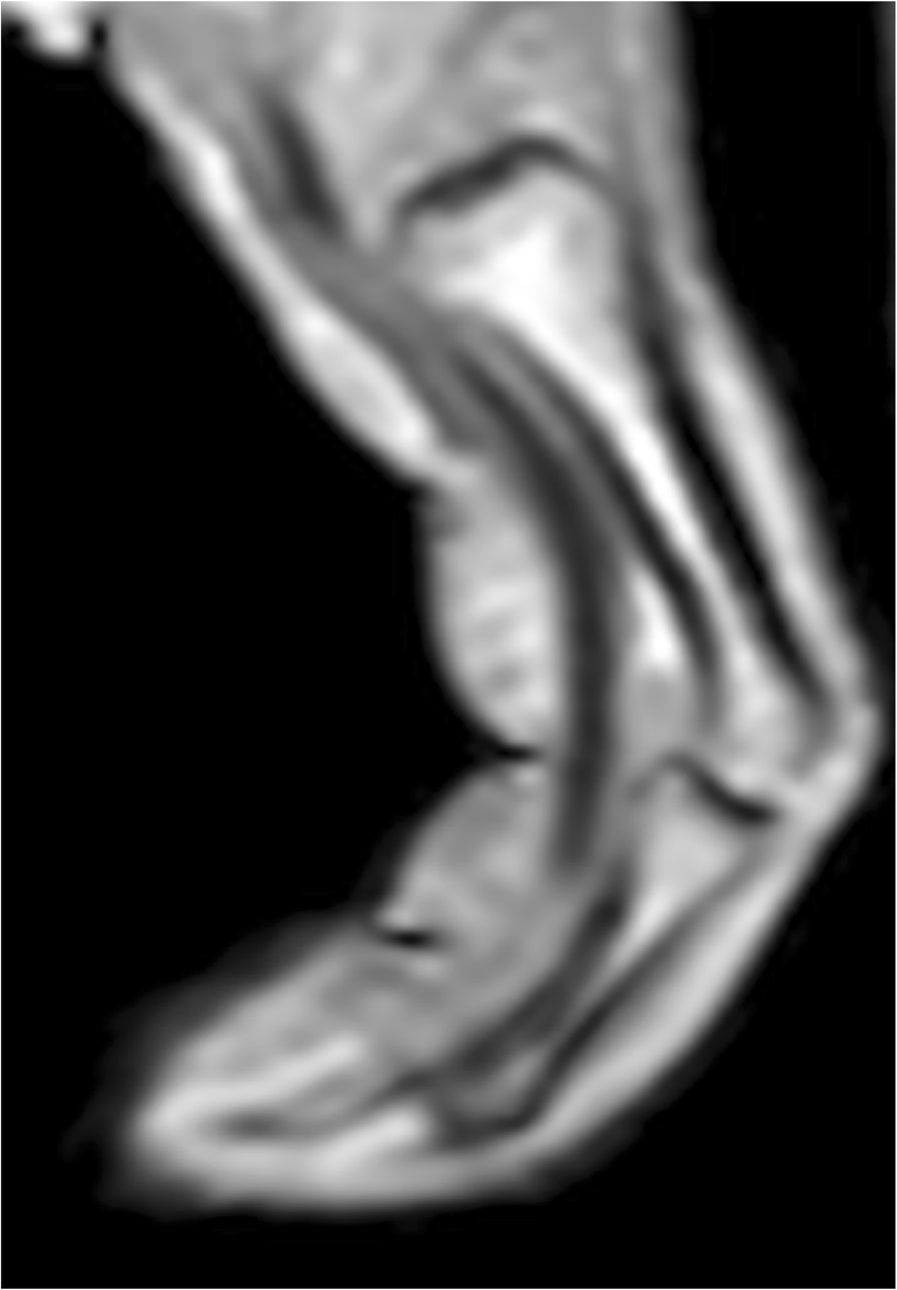
Real-time dynamic MR image obtained with the UFGE sequence at 3.0 T. Finger in the sagittal plane in flexion (TR, 4.7; TE, 2.3; flip angle, 15°; pixel size, 1.09 × 1.46 mm)

The selection of sequence parameters is a trade-off among temporal resolution, SNR, and spatial resolution. For example, Boutin et al. obtained a temporal resolution of 475 ms with a pixel size of 0.94 mm [33], whereas Pierrart et al. obtained a temporal resolution of 285 ms with a pixel size of 1.6 mm [28]. These sequences must be acquired with the shortest TR and TE to reduce acquisition time as well as the inhomogeneity due to T2* effects on balanced SSFP sequences [26]. A small flip angle improves SNR and provides a proton density-weighted image for RF-spoiled and UFGE sequences (Figs. 3 and 4, Movie 3, 4) [40, 42]. Cartesian and radial sampling of the *k*-space were both used for real-time dynamic MRI. Radial sampling was particularly suitable for real-time RF-spoiled sequences at 3.0 T [29, 35], allowing an increase in temporal resolution without image quality deterioration with the use of constrained iterative reconstruction [29]. Such iterative reconstruction of real-time images often requires the addition of graphic processing units to the current MRI systems [45, 46]. However, Cartesian sampling is more available and simpler to use for image reconstruction.

All slices covering the joint can be acquired during a single time frame by using sequential or multi-slice techniques [28, 35]. The total acquisition time will depend on the number of slices and time frames. For example, Boutin et al. obtained an acquisition time of 35 s with one slice and 60 time frames for a balanced SSFP sequence [33], whereas Clarke et al. obtained an acquisition time of 104 s with eight slices and 40 time frames for a UFGE sequence [37]. Parameters such as the number of slices, gap, and field of view depend on the studied joint, patient size, and slice orientation.

The acquisition plane must be adapted to the joint being imaged. For example, on the ankle, an axial plane is more appropriate to evaluate fibular tendon instability [47], and a sagittal plane is more appropriate to evaluate Achilles tendon motion (Fig. 3, Movie 3) [48].

## Patient and radiofrequency coil positioning in the MRI scanner

Optimal patient positioning in the scanner and RF coil positioning relative to the joint are fundamental for the accurate evaluation of joint motion with dynamic MRI. Joint and coil positioning inside the MRI scanner depends on the impairment and bone kinematics or tendon displacement to be explored (Table 3) (Movie 1, 2, 3 and 4).

**Table 3:** Clinical applications of joint dynamic MRI

Shoulder	Subacromial impingement [49, 50]
Wrist	Scapholunate instability [51]
	Extensor carpi ulnaris tendon instability [32]
Finger	Pulley injuries [52]
Hip	Femoroacetabular impingement [53]
Knee	Patellofemoral instability [16, 23, 43, 54]
	Anterior cruciate ligament deficiency [55–57]
	Post-traumatic medial laxity [36]
Ankle	Peroneal tendon subluxation [47]

The joint of interest must be positioned as centrally as possible inside the MRI bore to obtain a homogeneous field. Flex RF coils are often used for these acquisitions and must be positioned close to the joint for better signal homogeneity and SNR while preventing its displacement during joint motion. This may require specific fixtures to maintain the RF coil position close to the joint and to avoid contact with the body surface if necessary [26]. In most cases, cushions and devices provided by manufacturers should be effectively used without the need for additional fixtures [26, 32]. Joint positioning in the scanner requires a trade-off between the possible ROM and the necessity to be as close as possible to the RF coil to enhance the SNR. This primarily depends on the RF coil type and size that is available for each scanner. Joint motion may also be limited by its positioning within the MRI bore and by the bore size on closed-bore MRI scanners.

The incorporation of dynamic MRI acquisition in addition to the standard clinical MR exam with static sequences poses multiple challenges. The position of the patient or the RF coil should not be changed throughout the exam. Furthermore, the addition of the dynamic sequence should not substantially increase the total acquisition time. The RF coils used for real-time dynamic MRI make it possible to acquire standard static images with high image quality and thus can be used for both static and dynamic acquisitions at the same time and with the same setup [5, 28, 29, 37].

Considering these requirements, we have provided recommendations from the literature for setting up each joint type in the MRI scanner. For this purpose, we classified the joints into three groups for patient positioning and equipment setup description:

- Proximal joints (shoulder, hip)
- Intermediate joints (elbow, knee)
- Distal joints (wrist, hand, ankle, foot)

### Proximal joints

**Fig. 5:**
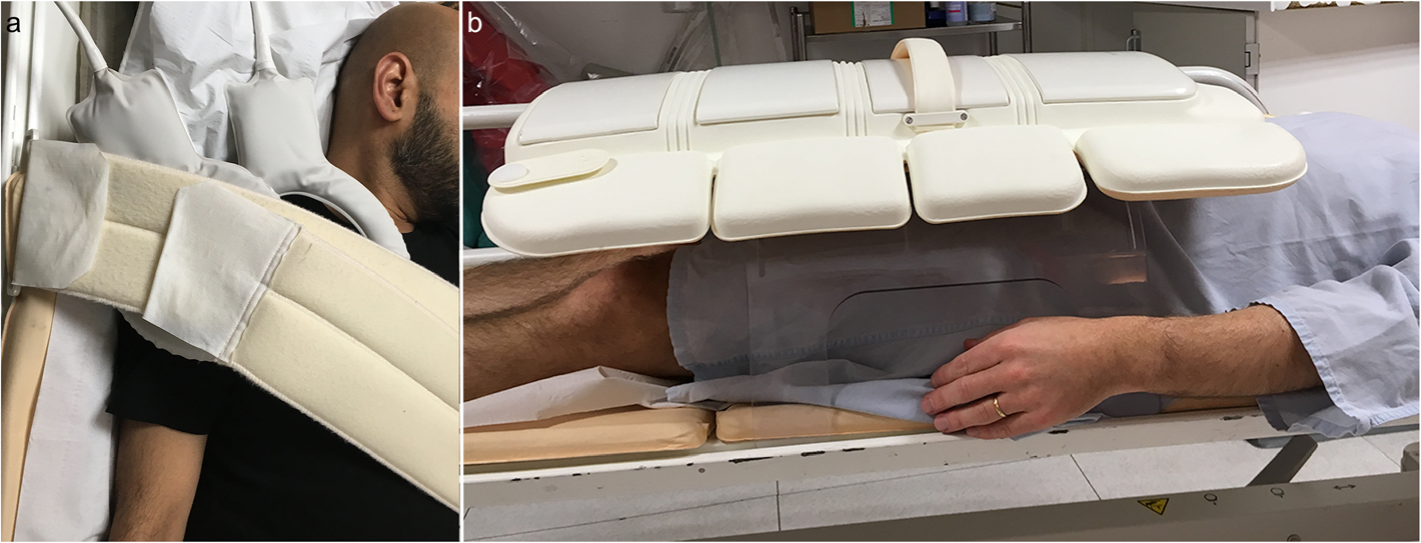
Patient and coil positioning for shoulder (**a**) and hip (**b**) motion evaluation in an Achieva dStream 3.0 T Philips MRI scanner

Dynamic imaging of proximal joints is challenging because these joints are hard to reach for RF coil positioning. The setup should be optimized for achieving maximal ROM within the MRI scanner bore. The patient is typically positioned supine, head-first into the bore for the shoulder examination and feet-first for the hip examination, with the joint close to the MR bore center, which allows more space for movement to occur [28]. For the shoulder examination, the arm is positioned along the patient’s side at rest [28, 58]. The coils are placed around the joint and could be maintained by a harness to avoid displacement during motion (Fig. 5, Movie 5) [58].

### Intermediate joints

**Fig. 6:**
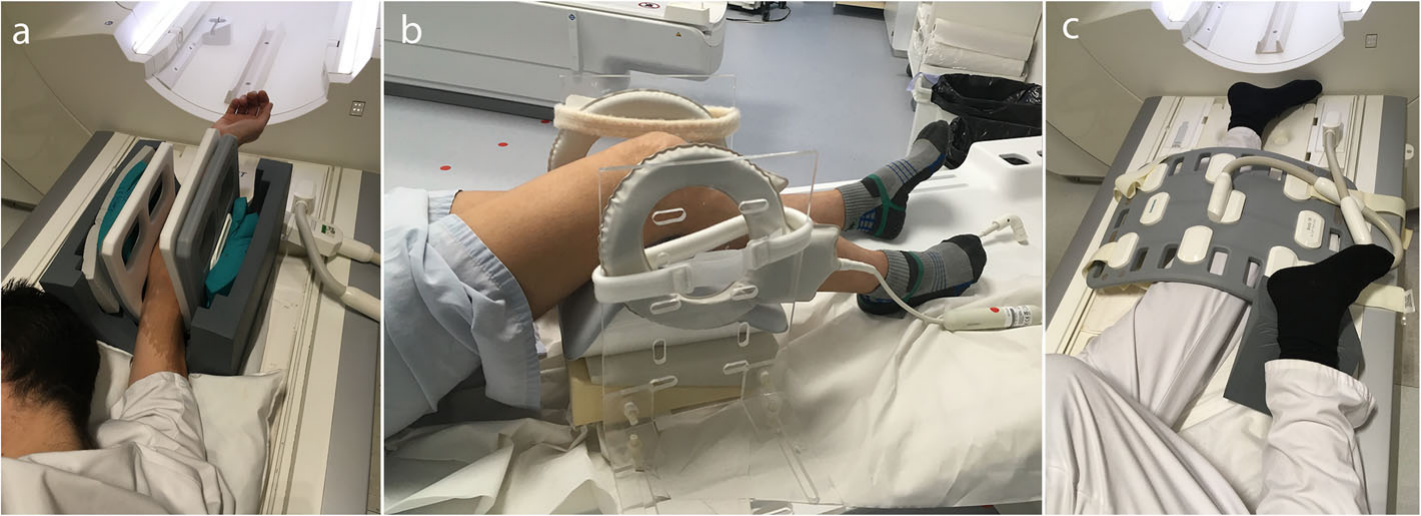
Patient and flex coil positioning for elbow (**a**) and knee motion evaluation (**b**, **c**). The flex coil is held by a dedicated support in an Avanto fit 1.5 T Siemens MRI scanner for elbow motion evaluation (**a**). The patient is positioned supine with a homemade support for coil positioning in an Achieva dStream 3.0 T Philips MRI scanner (**b**) or lateral decubitus in an Avanto fit 1.5 T Siemens MRI scanner (**c**) for knee motion evaluation

For the study of the elbow, the patient is positioned prone, head-first, arm above the head, with the elbow in the center of the RF coil which is held by a support (Fig. 6a) [26]. The kinematic study of the knee joint can be performed on a subject in a supine position, feet-first, with the flex coil held by a device around the joint (Fig. 6b) [59, 60]. The installation of a cushion under the knee increases the flexion/extension and degrees of freedom [5, 43], which are limited by the bore diameter and the size of the lower limb [61]. Unrestrained knee flexion/extension can also be achieved with the patient in side lying position and a large flex coil placed on the knee, and the other limb put on the coil to prevent its displacement (Fig. 6c) [44]. Some authors have also proposed prone positioning of the patient, allowing passive knee flexion [61] or knee flexion against resistance [18, 62].

### Distal joints

All degrees of motion of distal joints can be studied within the MRI scanner. For the wrist and hand, the patient is positioned prone, head-first, with the upper limb raised above the head. Langner et al. studied the abduction/adduction motion of the wrist with a flex coil positioned on the table parallel to the motion without any support [51].

**Fig. 7:**
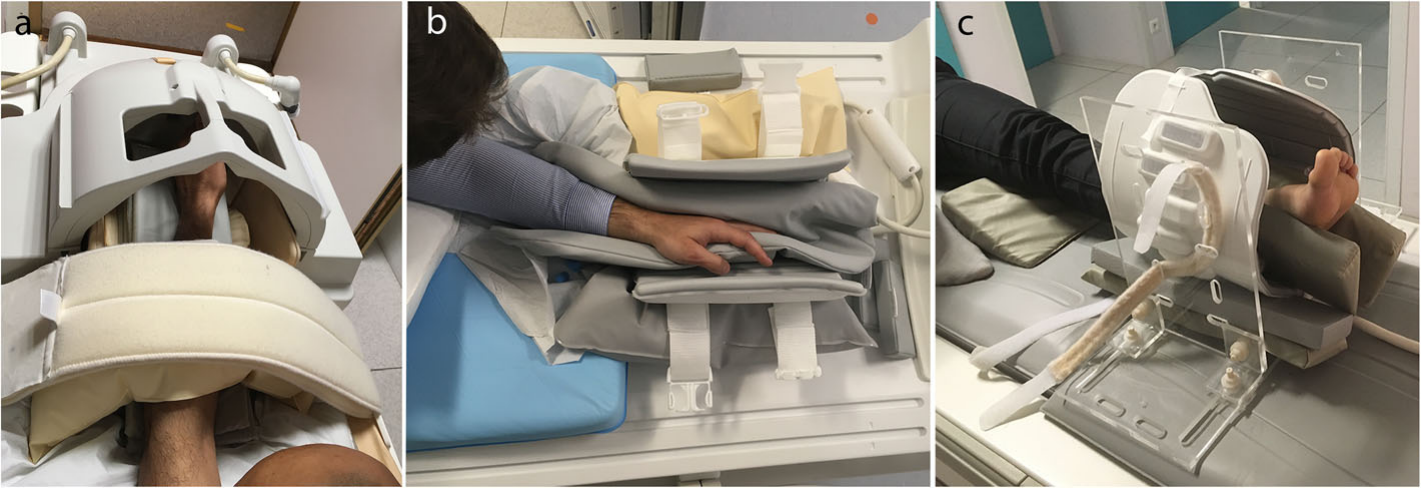
Patient and coil positioning for wrist (**a**), finger (**b**), and ankle motion evaluation (**c**). The wrist is positioned within a head coil in an Achieva dStream 3.0 T Philips MRI scanner (**a**). The hand is positioned within an extremity coil (which is opened to show positioning inside) for finger motion evaluation in an Achieva dStream 3.0 T Philips MRI scanner (**b**). The flex coil is positioned in the support of ankle motion evaluation in an Optima 1.5 T GE (**c**). Some cushions and sandbags are added to maintain the joint in the selected plane

The distal situation of these joints makes it possible to use a “rigid” RF coil. Bayer et al. performed dynamic MRI sequences of the finger with a sky boot-shaped coil [63], whereas Schellhammer and Vantorre used a knee coil to study finger motion [52]. The hand can also be positioned within a head coil to investigate all degrees of freedom (Fig. 7a) [29, 33], whereas Kaiser et al. used an extremity coil to explore wrist pronation-supination [32]. Given the difference between the coil size and the small joint volume, the use of pads or foams to hold the hand in a central position within the coil and to avoid undesired motion is required (Fig. 7b) [32].

For the ankle, the patient is in the supine position, and images can be obtained using a flex coil that is positioned around the joint with a device adapted to the RF coil model (Fig. 7c) [22, 24, 26].

## Limitations, solutions, and perspectives

Real-time dynamic MRI is based on high temporal resolution, but it also requires sufficient contrast, SNR, and spatial resolution for joint motion evaluations through image post-processing. The image quality also depends on the MRI scanner, magnetic field strength, and RF coil type [10]. Acquiring MRI at 3.0 T using flex coils with 16 channels can improve the acquisition time, SNR, and spatial resolution (Table 2).

However, these gradient echo sequences can be affected by some artifacts that worsen with higher magnetic field strength. Moreover, these sequences are susceptible to magnetic field inhomogeneities, resulting in signal loss and deformation. For example, a chemical shift artifact can appear as a black border at the fat/water interface. These artifacts can be reduced with a larger bandwidth, even if it decreases the SNR [33]. Chemical shift artifacts can also be reduced with in-phase TE [64].

Susceptibility and motion artifacts can be reduced with acceleration techniques such as parallel imaging with phased-array RF coils and partial or radial sampling of the *k*-space with iterative reconstructions [29, 65–68], which allow improvements in image quality and temporal resolution.

**Fig. 8:**
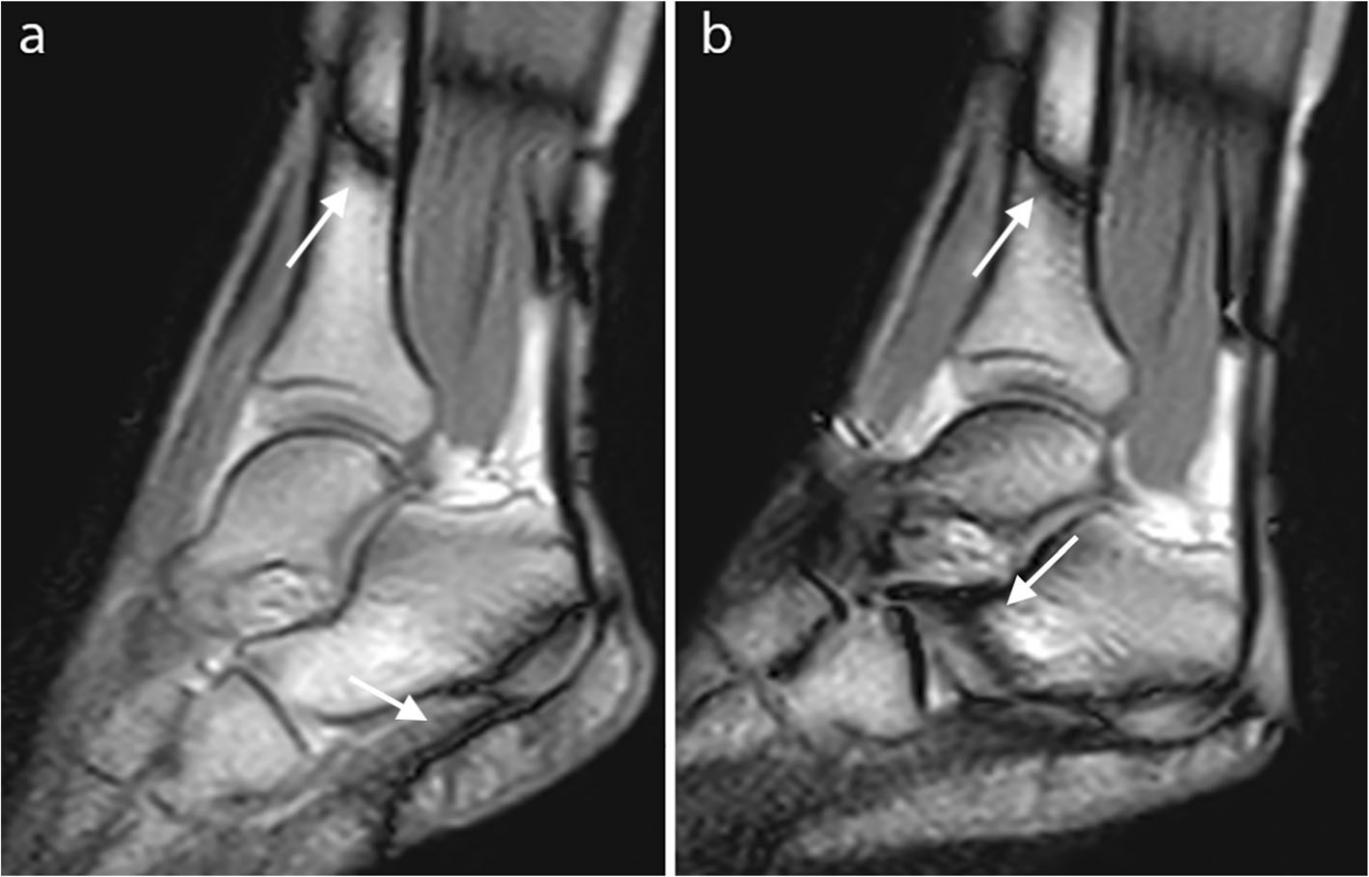
Sagittal balanced SSFP image of the ankle in plantar (**a**) and dorsal (**b**) flexion. Band artifacts appear as black lines that move on the image during motion

The balanced SSFP sequence is also deteriorated by band artifacts due to off-resonance effects from B_0_ non-uniformity, which are not present in RF-spoiled and UFGE sequences. These band artifacts can appear over the joint and disturb image analysis, in particular at 3.0 T (Fig. 8) [29, 45]. They can be reduced by minimizing TR or by using a 3D shim at the beginning of the examination [26, 64]. Other methods to reduce these band artifacts include the use of alternating TR, which widens the space between these bands [69]; multiple-offset method; frequency modulation [70]; or the use of specific algorithms [71]. Band artifacts can also be reduced by positioning the joint in an intermediate position during calibration, considering its ROM [26]. Field inhomogeneity is increased in the case of large difference in size between the joint and the RF coil, as for finger examination. Adding dielectric pads around the joint can improve the homogeneity of the field and reduce artifacts [33].

New post-processing methods are being developed to register images obtained during motion with high-resolution 3D static images. These methods make it possible to track bone motion in real-time [31, 37], to obtain 3D reconstructions of the bone structures during motion [34], and, therefore, to provide accurate quantitative biomechanical data.

## Conclusion

Three gradient echo sequences (balanced SSFP, RF-spoiled, and UFGE sequences) are available for real-time dynamic MRI of joints of the upper and lower limbs during continuous motion, considering their excellent temporal resolution, good SNR, and contrast. However, real-time dynamic MRI requires the adaptation of the sequence parameters, rigorous patient and coil positioning to allow an evaluation with a sufficient level of quality. These real-time sequences can be incorporated within a daily protocol for joint MR analysis due to their short acquisition time and would allow to better understand dynamic outcomes from specific joint disorders or to diagnose conditions not otherwise detected with static imaging. Future work may be focused on post-processing method integration in current exams to improve the SNR and to obtain 3D reconstruction while maintaining a short acquisition time.

## Supplementary information

**Additional file 1: Movie 1.** Balanced SSFP sequence of the shoulder in the axial plane during rotation at 3.0 T (same parameters as Fig. 1). This sequence shows glenohumeral rotation, subscapular tendon excursion and the contraction of the subscapularis and infraspinatus muscles during internal and external rotation, respectively.

**Additional file 2: Movie 2.** Balanced SSFP sequence of the wrist in the coronal plane during radial/ulnar abduction at 3.0 T (same parameters as Fig. 1). This sequence shows wrist bones relationship during motion.

**Additional file 3: Movie 3.** RF-spoiled sequence of the ankle in the sagittal plane during flexion at 3.0 T (same parameters as Fig. 3). This sequence allows us to investigate tibiotalar motion and Achilles tendon excursion.

**Additional file 4: Movie 4.** UFGE sequence of the finger in the sagittal plane during flexion at 3.0 T (same parameters as Fig. 4). This sequence allows the evaluation of bones motion and flexor tendon excursion.

**Additional file 5: Movie 5.** Shoulder motion in the three degrees of freedom after coil positioning.

## Data Availability

Not applicable

## Abbreviations

3D: Three-dimensional
CT: Computed tomography
MRI: Magnetic resonance imaging
RF: Radio frequency
ROM: Range of motion
SNR: Signal-to-noise ratio
SSFP: Steady-state free precession
TE: Echo time
TR: Repetition time
UFGE: Ultra-fast gradient echo

## References

[1] Tashman S, Collon D, Anderson K, Kolowich P, Anderst W (2004). Abnormal rotational knee motion during running after anterior cruciate ligament reconstruction. Am J Sports Med.

[2] Draper CE, Besier TF, Fredericson M (2011). Differences in patellofemoral kinematics between weight-bearing and non-weight-bearing conditions in patients with patellofemoral pain. J Orthop Res.

[3] Powers CM, Ward SR, Fredericson M, Guillet M, Shellock FG (2003). Patellofemoral kinematics during weight-bearing and non-weight-bearing knee extension in persons with lateral subluxation of the patella: a preliminary study. J Orthop Sports Phys Ther.

[4] McWalter EJ, O’Kane CM, FitzPatrick DP, Wilson DR (2014). Validation of an MRI-based method to assess patellofemoral joint contact areas in loaded knee flexion in vivo: patellofemoral contact area validation. J Magn Reson Imaging.

[5] d’Entremont AG, Nordmeyer-Massner JA, Bos C, Wilson DR, Pruessmann KP (2013). Do dynamic-based MR knee kinematics methods produce the same results as static methods?. Magn Reson Med.

[6] Carr R, MacLean S, Slavotinek J, Bain GI (2019). Four-dimensional computed tomography scanning for dynamic wrist disorders: prospective analysis and recommendations for clinical utility. J Wrist Surg.

[7] Guillin R, Marchand AJ, Roux A, Niederberger E, Duvauferrier R (2012). Imaging of snapping phenomena. Br J Radiol.

[8] Li G, Van de Velde SK, Bingham JT (2008). Validation of a non-invasive fluoroscopic imaging technique for the measurement of dynamic knee joint motion. J Biomech.

[9] Teixeira P, Gervaise A, Louis M (2015). Musculoskeletal wide-detector CT kinematic evaluation: from motion to image. Semin Musculoskelet Radiol.

[10] Shapiro LM, Gold GE (2012). MRI of weight bearing and movement. Osteoarthritis Cartilage.

[11] Sechtem U, Pflugfelder PW, White RD (1987). Cine MR imaging: potential for the evaluation of cardiovascular function. AJR Am J Roentgenol.

[12] van Dijk P (1984). ECG-triggered NMR imaging of the heart. Diagn Imaging Clin Med.

[13] Melchert UH, Schröder C, Brossmann J, Muhle C (1992). Motion-triggered cine MR imaging of active joint movement. Magn Reson Imaging.

[14] Burnett KR, Davis CL, Read J (1987). Dynamic display of the temporomandibular joint meniscus by using “fast-scan” MR imaging. AJR Am J Roentgenol.

[15] Zhu Y, Pelc NJ (1999). Three-dimensional motion tracking with volumetric phase contrast MR velocity imaging. J Magn Reson Imaging.

[16] Muhle C, Brossmann J, Melchert UH (1995). Functional MRI of the patellofemoral joint: comparison of ultrafast MRI, motion-triggered cine MRI and static MRI. Eur Radiol.

[17] Borotikar B, Lempereur M, Lelievre M, Burdin V, Ben Salem D, Brochard S (2017). Dynamic MRI to quantify musculoskeletal motion: a systematic review of concurrent validity and reliability, and perspectives for evaluation of musculoskeletal disorders. PLoS One.

[18] Shellock FG (2003). Functional assessment of the joints using kinematic magnetic resonance imaging. Semin Musculoskelet Radiol.

[19] Shellock FG, Mink JH, Deutsch AL, Fox JM (1989). Patellar tracking abnormalities: clinical experience with kinematic MR imaging in 130 patients. Radiology.

[20] Schmid MR, Hodler J, Cathrein P, Duewell S, Jacob HAC, Romero J (2002). Is impingement the cause of jumper’s knee? Dynamic and static magnetic resonance imaging of patellar tendinitis in an open-configuration system. Am J Sports Med.

[21] Scarvell JM, Smith PN, Refshauge KM, Galloway HR, Woods KR (2005). Association between abnormal kinematics and degenerative change in knees of people with chronic anterior cruciate ligament deficiency: a magnetic resonance imaging study. Aust J Physiother.

[22] Tokuda O, Awaya H, Taguchi K, Matsunga N (2006). Kinematic MRI of the normal ankle ligaments using a specially designed passive device. Foot Ankle Int.

[23] Brossmann J, Muhle C, Büll CC (1994). Evaluation of patellar tracking in patients with suspected patellar malalignment: cine MR imaging vs arthroscopy. AJR Am J Roentgenol.

[24] Sheehan FT, Seisler AR, Siegel KL (2007). In vivo talocrural and subtalar kinematics: a non-invasive 3D dynamic MRI study. Foot Ankle Int.

[25] Shellock FG, Foo TK, Deutsch AL, Mink JH (1991). Patellofemoral joint: evaluation during active flexion with ultrafast spoiled GRASS MR imaging. Radiology.

[26] Quick HH, Ladd ME, Hoevel M (2002). Real-time MRI of joint movement with trueFISP. J Magn Reson Imaging.

[27] Draper CE, Santos JM, Kourtis LC (2008). Feasibility of using real-time MRI to measure joint kinematics in 1.5 T and open-bore 0.5 T systems. J Magn Reson Imaging.

[28] Pierrart J, Lefèvre-Colau MM, Skalli W (2014). New dynamic three-dimensional MRI technique for shoulder kinematic analysis. J Magn Reson Imaging.

[29] Shaw CB, Foster BH, Borgese M (2019). Real-time three-dimensional MRI for the assessment of dynamic carpal instability. PLoS One.

[30] Yen P, Katzberg RW, Buonocore MH, Sonico J (2013). Dynamic MR imaging of the temporomandibular joint using a balanced steady-state free precession sequence at 3 T. AJNR Am J Neuroradiol.

[31] Gilles B, Perrin R, Magnenat-Thalmann N, Vallee JP (2005). Bone motion analysis from dynamic MRI: acquisition and tracking. Acad Radiol.

[32] Kaiser P, Kellermann F, Arora R, Henninger B, Rudisch A (2018). Diagnosing extensor carpi ulnaris tendon dislocation with dynamic rotation MRI of the wrist. Clin Imaging.

[33] Boutin RD, Buonocore MH, Immerman I (2013). Real-time magnetic resonance imaging (MRI) during active wrist motion—initial observations. PLoS One.

[34] Makki K, Borotikar B, Garetier M, Brochard S, Ben Salem D, Rousseau F (2019). In vivo ankle joint kinematics from dynamic magnetic resonance imaging using a registration-based framework. J Biomech.

[35] Henrichon SS, Foster BH, Shaw C (2020). Dynamic MRI of the wrist in less than 20 seconds: normal midcarpal motion and reader reliability. Skeletal Radiol.

[36] Studler U, White LM, Deslandes M, Geddes C, Sussman MS, Theodoropoulos J (2011). Feasibility study of simultaneous physical examination and dynamic MR imaging of medial collateral ligament knee injuries in a 1.5-T large-bore magnet. Skeletal Radiol.

[37] Clarke EC, Martin JH, d’Entremont AG, Pandy MG, Wilson DR, Herbert RD (2015). A non-invasive, 3D, dynamic MRI method for measuring muscle moment arms in vivo: demonstration in the human ankle joint and Achilles tendon. Med Eng Phys.

[38] Chavhan GB, Babyn PS, Jankharia BG, Cheng H-LM, Shroff MM (2008). Steady-state MR imaging sequences: physics, classification, and clinical applications. Radiographics.

[39] Bieri O, Scheffler K (2013). Fundamentals of balanced steady state free precession MRI. J Magn Reson Imaging.

[40] Hargreaves B (2012). Rapid gradient-echo imaging. J Magn Reson Imaging.

[41] Fuchs F, Laub G, Othomo K (2003). TrueFISP—technical considerations and cardiovascular applications. Eur J Radiol.

[42] Elster AD (1993). Gradient-echo MR imaging: techniques and acronyms. Radiology.

[43] Burke CJ, Kaplan D, Block T (2018). Clinical utility of continuous radial magnetic resonance imaging acquisition at 3 T in real-time patellofemoral kinematic assessment: a feasibility study. Arthroscopy.

[44] Fiorentino NM, Lin JS, Ridder KB, Guttman MA, McVeigh ER, Blemker SS (2013). Rectus femoris knee muscle moment arms measured *in vivo* during dynamic motion with real-time magnetic resonance imaging. J Biomech Eng.

[45] Lingala SG, Sutton BP, Miquel ME, Nayak KS (2016). Recommendations for real-time speech MRI. J Magn Reson Imaging JMRI.

[46] Frahm J, Voit D, Uecker M (2019). Real-time magnetic resonance imaging: radial gradient-echo sequences with nonlinear inverse reconstruction. Invest Radiol.

[47] VanPelt MD, Landrum MR, Igbinigie M, Wadhwa V, Chhabra A (2017). Kinematic magnetic resonance imaging of peroneal tendon subluxation with intraoperative correlation. J Foot Ankle Surg.

[48] Sheehan FT (2012). The 3D in vivo Achilles’ tendon moment arm, quantified during active muscle control and compared across sexes. J Biomech.

[49] Tempelaere C, Pierrart J, Lefèvre-Colau MM (2016). Dynamic three-dimensional shoulder MRI during active motion for investigation of rotator cuff diseases. PLoS One.

[50] Tasaki A, Nimura A, Nozaki T (2015). Quantitative and qualitative analyses of subacromial impingement by kinematic open MRI. Knee Surg Sports Traumatol Arthrosc.

[51] Langner I, Fischer S, Eisenschenk A, Langner S (2015). Cine MRI: a new approach to the diagnosis of scapholunate dissociation. Skeletal Radiol.

[52] Schellhammer F, Vantorre A (2019). Semi-dynamic MRI of climbing-associated injuries of the finger. Skeletal Radiol.

[53] Burke CJ, Walter WR, Gyftopoulos S (2019). Real-time assessment of femoroacetabular motion using radial gradient echo magnetic resonance arthrography at 3 Tesla in routine clinical practice: a pilot study. Arthroscopy.

[54] Muhle C, Brossmann J, Heller M (1999). Kinematic CT and MR imaging of the patellofemoral joint. Eur Radiol.

[55] Guenoun D, Vaccaro J, Le Corroller T (2017). A dynamic study of the anterior cruciate ligament of the knee using an open MRI. Surg Radiol Anat.

[56] Haughom BD, Souza R, Schairer WW, Li X, Benjamin Ma C (2012). Evaluating rotational kinematics of the knee in ACL-ruptured and healthy patients using 3.0 Tesla magnetic resonance imaging. Knee Surg Sports Traumatol Arthrosc.

[57] Barrance PJ, Williams GN, Snyder-Mackler L, Buchanan TS (2006). Altered knee kinematics in ACL-deficient non-copers: a comparison using dynamic MRI. J Orthop Res.

[58] Matsui K, Tachibana T, Nobuhara K, Uchiyama Y (2018). Translational movement within the glenohumeral joint at different rotation velocities as seen by cine MRI. J Exp Orthop.

[59] Borotikar Bhushan S., Sipprell William H., Wible Emily E., Sheehan Frances T. (2012). A methodology to accurately quantify patellofemoral cartilage contact kinematics by combining 3D image shape registration and cine-PC MRI velocity data. Journal of Biomechanics.

[60] Westphal CJ, Schmitz A, Reeder SB, Thelen DG (2013). Load-dependent variations in knee kinematics measured with dynamic MRI. J Biomech.

[61] Lin CC, Zhang S, Frahm J, Lu TW, Hsu CY, Shih TF (2013). A slice-to-volume registration method based on real-time magnetic resonance imaging for measuring three-dimensional kinematics of the knee. Med Phys.

[62] Powers CM, Shellock FG, Pfaff M (1998). Quantification of patellar tracking using kinematic MRI. J Magn Reson Imaging.

[63] Bayer T, Adler W, Janka R, Uder M, Roemer F (2017). Magnetic resonance cinematography of the fingers: a 3.0 Tesla feasibility study with comparison of incremental and continuous dynamic protocols. Skeletal Radiol.

[64] Huang SY, Seethamraju RT, Patel P, Hahn PF, Kirsch JE, Guimaraes AR (2015). Body MR imaging: artifacts, k-space, and solutions. Radiographics.

[65] Zaitsev M, Maclaren J, Herbst M (2015). Motion artifacts in MRI: a complex problem with many partial solutions. J Magn Reson Imaging.

[66] Deshmane A, Gulani V, Griswold MA, Seiberlich N (2012). Parallel MR imaging. J Magn Reson Imaging.

[67] Tsao J, Kozerke S (2012). MRI temporal acceleration techniques. J Magn Reson Imaging.

[68] Uecker Martin, Zhang Shuo, Voit Dirk, Merboldt Klaus-Dietmar, Frahm Jens (2012). Real-time MRI: recent advances using radial FLASH. Imaging in Medicine.

[69] Nayak KS, Lee HL, Hargreaves BA, Hu BS (2007). Wideband SSFP: alternating repetition time balanced steady state free precession with increased band spacing. Magn Reson Med.

[70] Foxall DL (2002). Frequency-modulated steady-state free precession imaging. Magn Reson Med.

[71] Björk M, Ingle RR, Gudmundson E, Stoica P, Nishimura DG, Barral JK (2014). Parameter estimation approach to banding artifact reduction in balanced steady-state free precession. Magn Reson Med.

